# Prognostic role of vitamin D receptor in digestive system tumours: A systematic review and preliminary meta-analysis

**DOI:** 10.1371/journal.pone.0289598

**Published:** 2023-08-10

**Authors:** Miaomiao Zhao, Zhenhua Liu, Hongtai Shi, Jianxiang Song

**Affiliations:** 1 Department of Ultrasound, The Yancheng Clinical College of Xuzhou Medical University, The First People’s Hospital of Yancheng, Yancheng, Jiangsu Province, China; 2 Department of Radiotherapy, The Yancheng Clinical College of Xuzhou Medical University, The First People’s Hospital of Yancheng, Yancheng, Jiangsu Province, China; 3 Department of Radiation Oncology, The Sixth Affiliated Hospital of Nantong University, Yancheng Third People’s Hospital, Yancheng, Jiangsu Province, China; 4 Department of Cardiothoracic Surgery, The Sixth Affiliated Hospital of Nantong University, Yancheng Third People’s Hospital, Yancheng, Jiangsu Province, China; Affiliated Hospital of Nanjing University of Chinese Medicine: Jiangsu Province Academy of Traditional Chinese Medicine, CHINA

## Abstract

The prognostic value of vitamin D receptor (VDR) in a variety of digestive system tumours remains controversial. In view of this, we conducted a meta-analysis. Published studies (as of Mar 30, 2023) assessing the prognostic role of VDR in digestive system tumours were retrieved. Pooled analyses were conducted based on the hazard ratios (HRs) of high VDR expression extracted from the included studies. If heterogeneity was detected, the random-effects model was used; otherwise, the fixed-effects model was used. Subgroup analysis, sensitivity analysis and meta-regression were performed to explore the sources of heterogeneity. Eight studies with 3,109 patients were included. The pooled results indicated that patients with high VDR expression generally had better overall survival (OS) (pooled HR = 0.67; 95% CI = 0.53–0.85; *P* = 0.001). Subgroup analyses showed that tumour type was the variable affecting the association between VDR expression and OS. VDR expression in colorectal cancer was not associated with OS (pooled HR = 0.84; 95% CI = 0.68–1.03; *P* = 0.086). We eliminated publication bias using the “trim and fill” method and found that high VDR expression remained an indicator of good OS (*P* = 0.001). Only a few studies explored the relationship between VDR expression and cancer-specific survival (CSS) or progression-free survival (PFS), and the pooled results indicated no association between them (*P*>0.05). VDR expression is a prognostic indicator in digestive system tumours and may also be used as a reference for vitamin D supplementation. Detection of VDR expression not only helps to evaluate prognosis but also to formulate more precise treatment plans for patients with digestive system tumours.

## Introduction

Vitamin D receptor (VDR) is an intranuclear biological macromolecule that mediates 1,25(OH)_2_D_3_ to exert biological effects [1]. Many of the biological functions of vitamin D are achieved through target gene transcription regulated by VDR. 1,25(OH)_2_D_3_ binds to VDR in target cells to form a hormone-receptor complex, which acts on specific DNA sequences of target genes to regulate their expression [2]. VDR is essentially a ligand-dependent nuclear transcription factor that plays an important role in maintaining calcium-phosphorus metabolism and regulating cell proliferation and differentiation [3]. VDR is widely distributed in normal tissue cells in the body. In addition, VDR is found in some tumour tissues, such as breast cancer [4, 5], oesophageal cancer [6], and colorectal cancer [7]. In 2020, we conducted a meta-analysis to evaluate the prognostic value of VDR expression in breast cancer [8]. In the literature search, we found that the prognostic value of VDR expression has been studied in a variety of tumours other than breast cancer, especially in digestive system tumours, such as oesophageal cancer, colorectal cancer, and pancreatic cancer [9, 10]. Therefore, we sought to determine the prognostic role of VDR in digestive system tumours.

The WHO (2019) classification of digestive system tumours includes a total of 12 categories, as follows: oesophagus, stomach, small intestine and ampulla, appendix, colon and rectum, duct, liver and intrahepatic bile duct, gallbladder and extrahepatic bile duct, pancreas, lymphoid haematopoietic system tumours, mesenchymal tumours, and other tumours [11]. Based on this, we screened published studies related to VDR expression and prognosis of malignant tumours and included eight related studies on digestive system tumours (three on colorectal cancer, three on oesophageal cancer, one on pancreatic cancer, and one on cholangiocarcinoma) [6, 7, 9, 10, 12–16]. This study aimed to meta-analyse published studies on VDR and prognosis to verify the role of VDR in the prognosis of patients with digestive system tumours.

The incidence of digestive system tumours ranks first in the world for malignant tumours and is also the main cause of cancer-related death [17]. In recent years, screening and treatment techniques for digestive system tumours have rapidly developed, but the overall survival of patients has not significantly improved [18]. Therefore, it is still necessary to find new biomarkers to monitor the condition of patients with digestive system tumours. Of the eight studies included, only two indicated that VDR expression in digestive system tumour tissues was associated with prognosis [6, 13]; the others indicated no association. The present meta-analysis, which pooled these disparate results, preliminarily showed that VDR expression is associated with overall survival in digestive system tumours. Accordingly, VDR expression is a potential prognostic indicator of digestive system tumours.

## Methods

### Guidelines and registration

This meta-analysis was undertaken according to Systematic Reviews and Meta-Analyses (PRISMA) guidelines [19]. The protocol for this review was registered in PROSPERO (CRD42023401849).

### Eligibility criteria

The inclusion criteria were as follows: (1) observational and clinical prospective and retrospective studies investigating the prognostic role of VDR in patients with digestive system tumours; (2) patients pathologically diagnosed with digestive system tumours; (3) hazard ratios (HRs) and 95% confidence intervals (CIs) or survival curves provided. The exclusion criteria were as follows: (1) reviews, letters, case reports, animal trials and conference abstracts; (2) for multiple studies with follow up of the same group of patients, the most complete or latest one was included. These criteria refer to those used in our previous study [8].

### Information sources and search strategy

Published studies (as of Mar 30, 2023) from PubMed, Embase, and Cochrane Library were retrieved. No language restrictions were applied. Three medical subject heading (MeSH) terms (“receptors, calcitriol”, “carcinoma”, and “neoplasms”) and their synonyms together constituted the search strategy. The details of the search strategy are listed in S1 Table.

### Study selection

The selection followed the PRISMA 2009 flow diagram [20]. Studies identified through database searching or other sources were initially screened by reading abstracts after duplicates were removed. The full text of the remaining studies was read for further elimination. Then, the quality of each study was scored according to the Newcastle‒Ottawa Quality Assessment Scale (NOS) on a scale from 0 to 9. Finally, studies with qualified quality (score ≥ 6) assessments were included. Each selection process was carried out independently by two authors, and differences were resolved through discussion.

### Data collection and data items

The methods used for data extraction also refer to those in our previous study [8]. In one study, the results of multivariate analysis were preferred to those of univariate analysis. If the study did not directly report HR and only provided survival curves, we estimated HR from the curves according to Tierney’s method [21].

### Statistical analysis

At a *P* value of the chi-square test ≥ 0.1 and *I*^2^ < 50%, the heterogeneity between studies was not significant, and the fixed-effects model (the Mante-Haenszel method) was applied [22]. Otherwise, the random-effects model (the DerSimonian‒Laird method) was used [23]. Subgroup analysis, sensitivity analysis and meta-regression were performed to explore the sources of heterogeneity. Publication bias was assessed according to the visual symmetry of funnel plots. Begg’s and Egger’s tests were performed to quantitatively assess publication bias. If publication bias was detected, the Duval and Tweedie trim-and-fill method was used to adjust the bias [24]. STATA version 12.0 (Stata Corporation, College Station, TX, USA) was applied to analyse data and generate figures. Except for estimating heterogeneity and publication bias, a *P* value less than 0.05 was considered significant.

## Results

### Study characteristics

**Fig 1:**
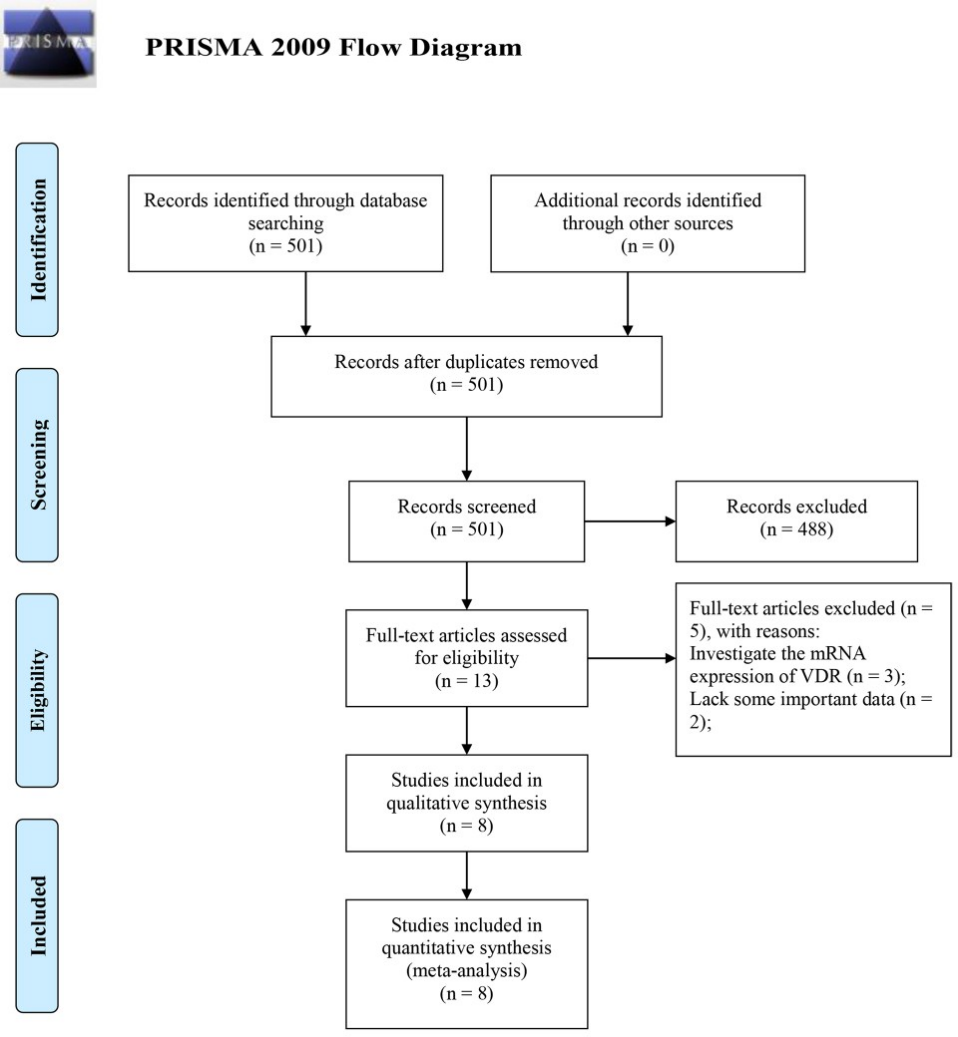
Flow diagram of the study selection process for the meta-analysis.

Fig 1 shows the retrieval process. Initially, 501 studies were identified. After excluding 488 studies not reporting the prognostic value of VDR, the remaining studies were read in detail. Among them, three studies were excluded because only mRNA expression of VDR was detected, and two other studies were excluded because of a lack of important data. Finally, eight studies with 3,109 patients were included in this meta-analysis (Fig 1) [6, 7, 9, 10, 12–16]. Their NOS scores ranged from 6 to 8, with an average score of 7.375 (Table 1).

**Table 1:** Main characteristics of all studies included in the meta-analysis.

Study	Tumour type	Country	Case number	High expression (%)	Pathological type	Molecular type	Neoadjuvant treatment	Tumour stage	Detection method	Cut-off value	Multivariate analysis	HRs provided from	Outcome	NOS score
Shi 2020 [12]	Colorectal	China	188	80 (53.2)	Adenocarcinoma	Multiple	No	110/78(I-II/III-IV)	IHC	IRS>3	Yes	Report	OS	7
Wang 2015 [10]	Pancreatic	China	981	585 (59.6)	Adenocarcinoma	Multiple	NR	595/263(I-IIA/IIB-III)	IHC	IRS>3	Yes	Report	OS	8
Chiang 2017 [13]	Cholangiocarcinoma	China	82	45 (54.9)	Adenocarcinoma	Multiple	NR	NR	IHC	IRS>3	Yes	Report	OS	6
Bao 2018 [6]	Oesophageal	China	362	72 (19.9)	Squamous cell carcinoma	Multiple	NR	NR	IHC	NR	No	SC	OS	8
McCain 2018 [9]	Oesophageal	Northern Ireland	130	68 (52.3)	Adenocarcinoma	Triple negative	Yes	NR	IHC	Median H-score	Yes	Report	OS/CSS	7
Mayorga 2017 [14]	Colorectal	Spain	658	168 (25.5)	Adenocarcinoma	NR	No	IV	IHC	Optimal value	Yes	Report	OS/PFS	8
Kure 2009 [15]	Colorectal	America	599	225 (37.6)	Adenocarcinoma	Multiple	NR	NR	IHC	H-score≥50	Yes	Report	OS/CSS	8
Zhou 2014 [16]	Oesophageal	America	109	89 (78.9)	Adenocarcinoma	Multiple	No	30/52/17/10(I/II/III/IV)	IHC	H-score≥20	No	SC	OS	7

NR: not reported; IRS: immunoreactive score; IHC: immunohistochemistry; HR: hazard ratio; SC: survival curve; OS: overall survival; CSS: cancer-specific survival; PFS: progression-free survival; NOS: Newcastle‒Ottawa Quality Assessment Scale.

The important clinical parameters extracted are shown in Table 1. The digestive system tumours investigated included colorectal cancer in three studies, oesophageal cancer in three studies, pancreatic cancer in one study, and cholangiocarcinoma in one study. Patients were from four countries: China, Northern Ireland, Spain, and America. Patients in only one study received neoadjuvant treatment [9]. The pathological type in only one study was squamous cell carcinoma [6], with adenocarcinoma accounting for the remainder. All included studies used immunohistochemistry (IHC) to detect VDR expression. Six studies applied multivariate analysis [9, 10, 12–15].

### Overall survival (OS)

**Fig 2:**
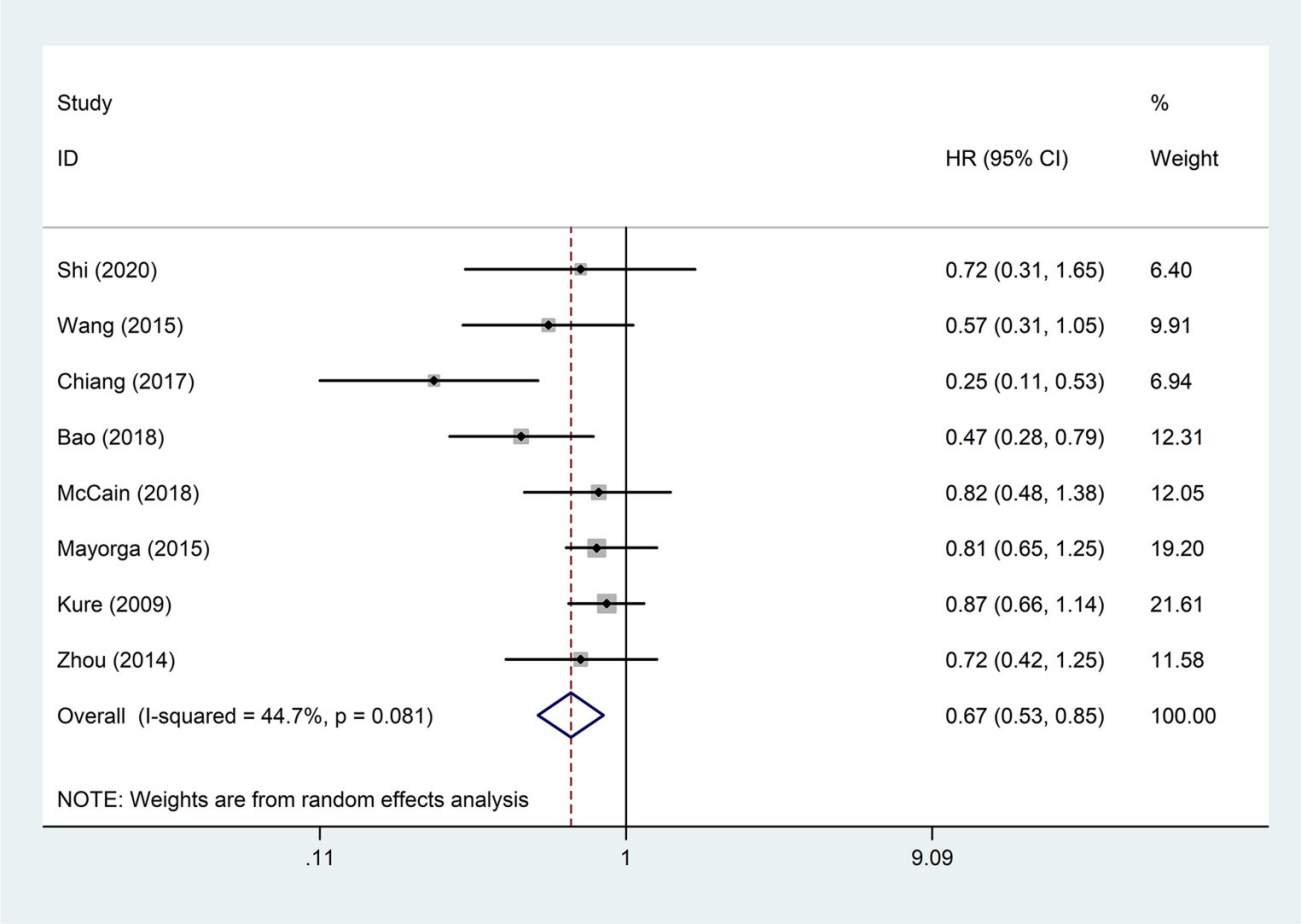
Forest plot of studies evaluating the hazard ratio of high VDR expression for overall survival of patients with digestive system tumours. VDR: vitamin D receptor; HR: hazard ratio; CI: confidence interval.

The correlation between VDR expression and OS of digestive system tumours was investigated in eight studies involving 3,109 patients [6, 9, 10, 12–16]. The random-effects model was applied for pooled analyses because of significant heterogeneity between these studies (*I*^2^ = 44.7%, *P* = 0.081). The pooled results showed that patients with high VDR expression had better OS (pooled HR = 0.67; 95% CI = 0.53–0.85; *P* = 0.001) (Table 2 and Fig 2).

**Table 2:** Pooled associations between VDR expression and patient prognosis.

Outcome subgroup	Study number	Case number	HR (95% CI)-model	*P* value	Heterogeneity	
					*I*^2^ (%)	*P*
OS	8	3,109	0.67 (0.53–0.85)-random	0.001	44.7	0.081
Tumour type						
Colorectal	3	1,445	0.84 (0.68–1.03)-fixed	0.086	0	0.886
Oesophageal	3	601	0.65 (0.48–0.88)-fixed	0.006	16.0	0.304
Others	2	1,063	0.39 (0.18–0.88)-random	0.023	62.0	0.105
Tumour location						
Digestive tract	6	2,046	0.77 (0.65–0.92)-fixed	0.003	0	0.483
Digestive gland	2	1,063	0.39 (0.18–0.88)-random	0.023	62.0	0.105
Race						
Asian	4	1,613	0.48 (0.34–0.66)-fixed	<0.001	22.6	0.275
Caucasian	4	1,496	0.83 (0.69–0.99)-fixed	0.042	0	0.941
Pathological type						
Adenocarcinoma	7	2,747	0.76 (0.64–0.90)-fixed	0.001	38.3	0.136
Squamous cell carcinoma	1	362	0.47 (0.28–0.79)	0.004	-	-
Cut-off value						
IRS > 3	3	1,251	0.48 (0.32–0.73)-fixed	0.001	48.3	0.144
Others	5	1,858	0.78 (0.65–0.92)-fixed	0.004	10.0	0.349
CSS	2	729	0.86 (0.64–1.17)-fixed	0.343	0	0.861
PFS	1	658	0.94 (0.75–1.43)	0.707	-	-

VDR: vitamin D receptor; OS: overall survival; IRS: immunoreactive score; CSS: cancer-specific survival; PFS: progression-free survival; HR: hazard ratio; CI: confidence interval.

**Fig 3:**
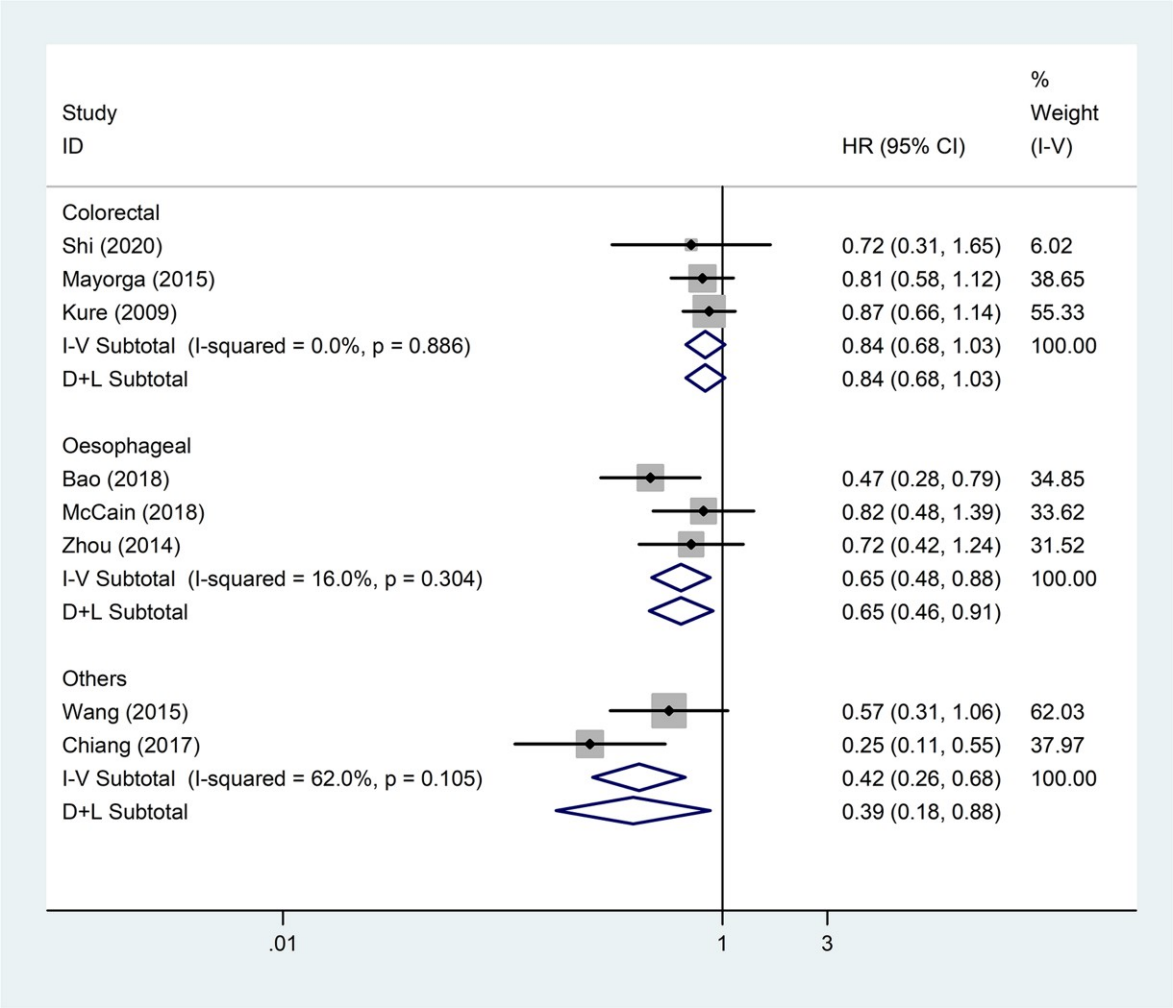
Forest plot of studies evaluating the hazard ratio of high VDR expression for overall survival of patients with digestive system tumours stratified by tumour type. VDR: vitamin D receptor; HR: hazard ratio; CI: confidence interval.

Subgroup analyses were performed according to tumour type, tumour location, race, pathological type, and cut-off value. The results showed that tumour location, race, pathological type, and cut-off value did not affect the association between VDR expression and patient OS but that tumour type did (*P*<0.05, Table 2). VDR expression in colorectal cancer was not related to OS (pooled HR = 0.84; 95% CI = 0.68–1.03; *P* = 0.086) (Table 2 andeq Fig 3).

**Fig 4:**
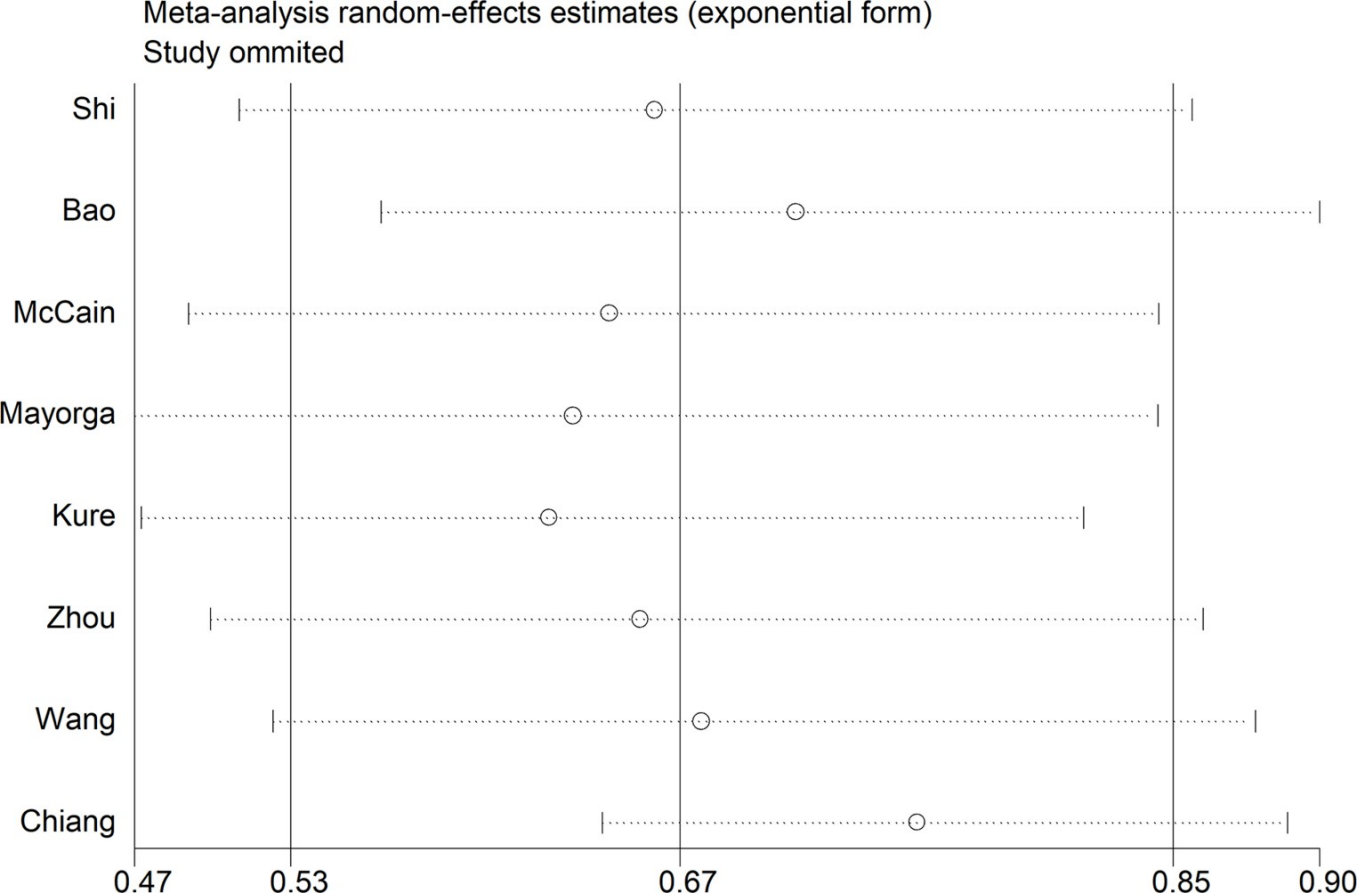
Sensitivity analysis of studies evaluating the relationship between VDR expression and overall survival in patients with digestive system tumours. VDR: vitamin D receptor; CI: confidence interval.

The results of sensitivity analysis showed that exclusion of any single study did not obviously eliminate the observed heterogeneity (Fig 4). Meta-regression results showed that tumour type (*P* = 0.533), tumour location (*P* = 0.425), race (*P* = 0.693), pathological type (*P* = 0.442), and cut-off value (*P* = 0.614) were not sources of heterogeneity.

**Fig 5:**
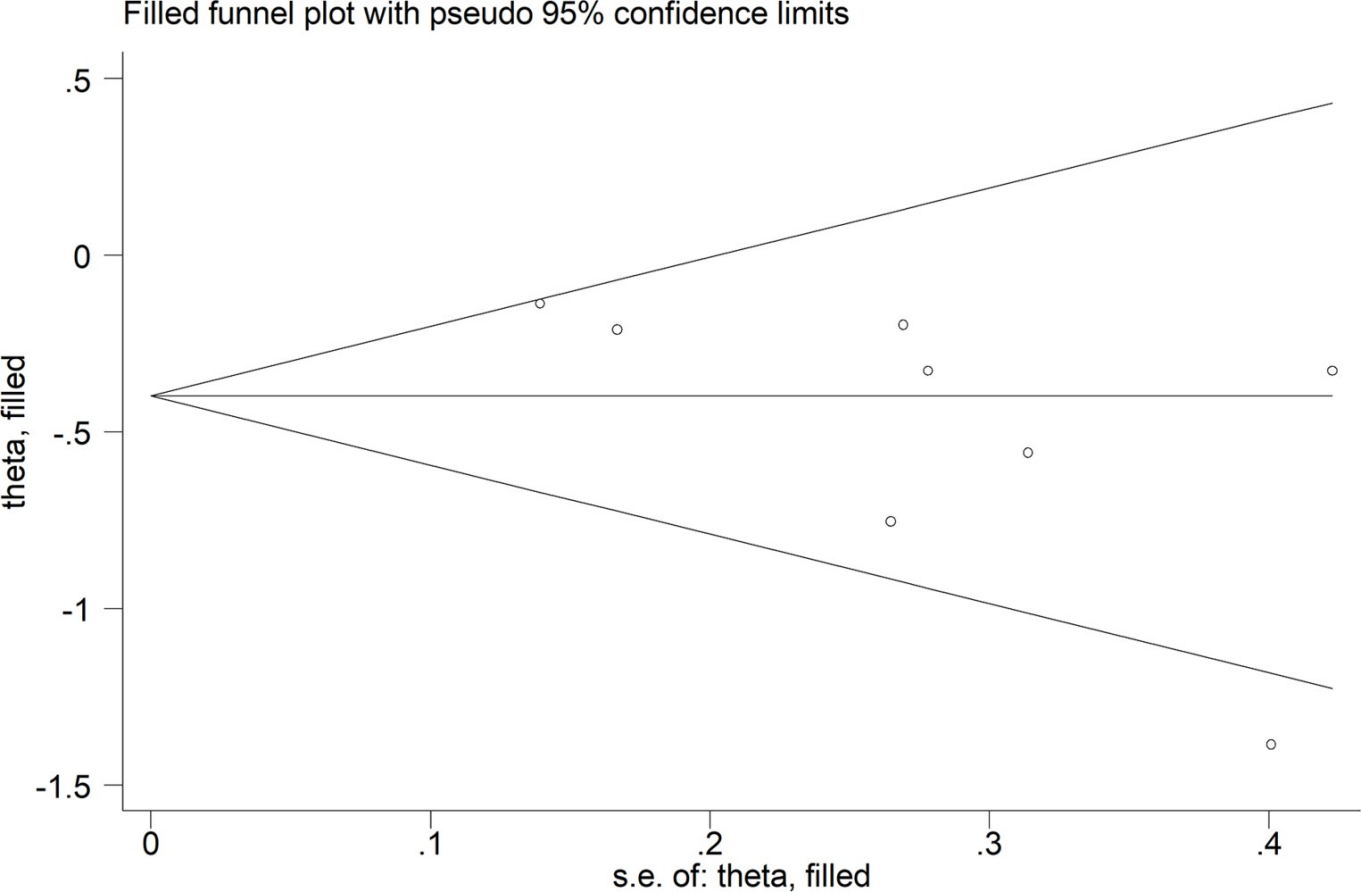
Funnel plot of publication bias for studies evaluating the relationship between VDR expression and patient overall survival in digestive system tumours.

Because the funnel plot of Begg’s test was asymmetrical (Fig 5) and the *P* value of Egger’s tests was less than 0.05, publication bias existed. We eliminated publication bias using the “trim and fill” method and found that high VDR expression was still an indicator of good OS (corrected pooled HR = 0.67; 95% CI = 0.53–0.85; *P* = 0.001).

### Cancer-specific survival (CSS) and progression-free survival (PFS)

Only two studies reported the association between VDR expression and CSS in patients with digestive system tumours [9, 15]: one related to oesophageal cancer, which included 130 patients [9], and another on colorectal cancer, which included 599 patients [15]. As there was no heterogeneity between these two studies (*I*^2^ = 0%, *P* = 0.861), a fixed-effects model was applied. Pooled results preliminarily indicated that VDR expression may not be related to CSS (pooled HR = 0.86; 95% CI = 0.64–1.17; *P* = 0.343) (Table 2). Only one study [14] including 658 patients with colorectal cancer noted that VDR expression did not correlate with patient PFS (HR = 0.94; 95% CI = 0.75–1.43) (Table 2).

## Discussion

Our meta-analysis included clinical information for 3,109 patients with digestive system tumours from eight independent studies. The results preliminarily suggested that patients with high VDR expression have better OS than patients with low VDR expression. VDR expression can be used as a supplement to tumour node metastasis (TNM) staging of digestive system tumours. However, subgroup analyses indicated that VDR is not associated with OS in patients with colorectal cancer. The insufficient sample size may be one of the reasons for this difference. In addition, the biological functions of VDR in different digestive system tumours differ, which may be another more important reason.

The biological functions of VDR have been investigated in colorectal cancer. For example, Shi et al. [12] found that VDR expression in colorectal cancer tissues was lower than that in adjacent tissues. Another study confirmed that VDR expression is associated with the PI3K-AKT pathway and KRAS mutation in colorectal cancer [15]. Binding of vitamin D and its receptor can reduce susceptibility to precancerous diseases such as inflammatory bowel disease [25, 26]. Moreover, VDR can upregulate the expression of the tumour-suppressor gene E-cadherin by targeting the Wnt/β-catenin pathway [25–27]. Given the above, high expression of VDR in colorectal cancer should predict better OS. However, subgroup analysis in this study showed that VDR expression was not associated with OS in colorectal cancer. Perhaps expanding the sample size will lead to more precise conclusions.

In oesophageal squamous cell carcinoma, the biological functions of VDR may be associated with the JNK1 pathway. Bao et al. [6] reported that expression levels of VDR and JNK1 were reduced in cancer tissues. They proposed that VDR functions with JNK1 in oesophageal epithelial cells and the stroma to inhibit tumorigenesis and metastasis by suppressing STAT3/AKT and EMT-related proteins [6]. However, the biological functions of VDR in oesophageal adenocarcinoma are not clear. Although some of the studies included in this meta-analysis indicated that VDR expression is not related to prognosis of oesophageal cancer, the pooled result indicated high VDR expression to be associated with better OS.

In addition, the synthetic analogues of vitamin D activate the VDR activator to arrest the pancreatic cancer cell cycle at G1 by downregulating expression of both β-catenin and AKT [10, 28]. The antiproliferative potency of these analogues depends on the level of VDR in cells [29]. For cholangiocarcinoma, vitamin D analogues can arrest the cell cycle at G0/G1 phase and inhibit human neutrophil gelatinase-associated lipocalin (NGAL) to repress growth of cholangiocarcinoma cells *in vitro* and *in vivo* [30, 31]. Induction of G0/G1 cell cycle arrest in cholangiocarcinoma cells by vitamin D analogues is mediated by upregulation of p27 and downregulation of CDK4, CDK6, and cyclin D3. Both pancreatic cancer and cholangiocarcinoma are digestive gland tumours. The pooled result showed that VDR is a good prognostic factor in digestive gland tumours, consistent with its biological function. More studies with large sample sizes are needed for confirmation.

In addition to VDR expression, serum vitamin D levels can be used to evaluate prognosis of patients with digestive system tumours [32, 33]. However, serum vitamin D levels are easily affected by diet and sun exposure [8]. On the other hand, evaluating the expression level of VDR is beneficial for formulating appropriate treatment plans for patients with digestive system tumours. An observational study investigated the effect of postoperative vitamin D supplementation on the prognosis of patients undergoing surgical resection of oesophageal squamous cell carcinoma [33]. A total of 280 postoperative patients were included, 49 of whom took 200–400 international units of vitamin D supplements daily [33]. The results showed that vitamin D supplementation did not improve overall survival but that it reduced the disease recurrence rate by 39% (HR = 0.61; 95% CI = 0.38–0.98) [33].

Our meta-analysis is the first to study the prognostic value of VDR in digestive system tumours. Some shortcomings need to be pointed out. First, digestive system tumours include 12 major categories, and there is heterogeneity among tumour types. However, due to the insufficient number of included studies, we only divided three subgroups according to tumour type, which would lead to insufficient representativeness of the results. Second, the number of included studies investigating patient CSS or PFS was too small to reach reliable results. Third, various cut-off values were used in the included studies, and the cut-off value is a factor affecting the prognostic value of VDR. Although the subgroup analysis of cut-off values indicated VDR to be a favourable prognostic factor both when using “IRS > 3” as the cut-off value and when using other cut-off values, a unified cut-off value to distinguish high and low VDR expression still needs to be established.

## Conclusions

We demonstrate that in general, VDR expression is a prognostic indicator for digestive system tumours. The association between VDR expression and prognosis was not impacted by tumour location, race, pathological type, or cut-off value, though the predictive effect of VDR seems more significant in oesophageal cancer. The role of prognostic evaluation supports to a certain extent that VDR can be used as a therapeutic target. Vitamin D analogues bind to VDR in tumour cells and activate downstream pathways to inhibit tumour growth. Perhaps intake of vitamin D analogues should be determined according to VDR expression. Therefore, by detecting VDR expression, oncologists can not only better evaluate the prognosis of patients with digestive system tumours but also formulate more precise treatment plans.

## Supporting information

S1 Table Details of the search strategy. No.: number; MeSH: medical subject heading.
(DOCX)
