# Supplementary material for: Prognostic role of vitamin D receptor in digestive system tumours: A systematic review and preliminary meta-analysis
Source: PLoS One. 2023 Aug 10;18(8):e0289598. doi: 10.1371/journal.pone.0289598 (PMC10414644; doi:10.1371/journal.pone.0289598)
Supplement: S1 Table — No.: number; MeSH: medical subject heading. (DOCX) [file pone.0289598.s001.docx]

**S1 Table. Details of the search strategy.**

| **No.** | **Search items** |
| --- | --- |
| [#1](https://www.ncbi.nlm.nih.gov/pubmed/advanced) | “receptors, calcitriol”[MeSH Terms] OR (“receptors”[All Fields] AND “calcitriol”[All Fields]) OR “calcitriol receptors”[All Fields] OR “vitamin D receptor”[All Fields] OR (“VDR”[All Fields]) |
| [#2](https://www.ncbi.nlm.nih.gov/pubmed/advanced) | “carcinoma”[MeSH Terms] OR “carcinoma”[All Fields] OR “carcinomas”[All Fields] OR “neoplasms”[MeSH Terms] OR “neoplasms”[All Fields] OR “neoplasm”[All Fields] OR “cancer”[All Fields] OR “cancers”[All Fields] OR “tumor”[All Fields] OR “tumors”[All Fields] OR “tumour”[All Fields] OR “tumours”[All Fields] |
| [#3](https://www.ncbi.nlm.nih.gov/pubmed/advanced) | [#1](https://www.ncbi.nlm.nih.gov/pubmed/advanced) and [#2](https://www.ncbi.nlm.nih.gov/pubmed/advanced) |

No.: number; MeSH: medical subject heading.
